# Detection of Three *Sarcocystis* Species (Apicomplexa) in Blood Samples of the Bank Vole and Yellow-Necked Mouse from Lithuania

**DOI:** 10.3390/life14030365

**Published:** 2024-03-10

**Authors:** Petras Prakas, Naglis Gudiškis, Neringa Kitrytė, Dovilė Laisvūnė Bagdonaitė, Laima Baltrūnaitė

**Affiliations:** Nature Research Centre, Akademijos Str. 2, 08412 Vilnius, Lithuania; naglis.gudiskis@gmail.com (N.G.); neringa.kitryte@gamtc.lt (N.K.); bagdonaitedl@gmail.com (D.L.B.); laima.baltrunaite@gamtc.lt (L.B.)

**Keywords:** *Sarcocystis*, rodents, blood, genetic identification, prevalence, *cox1*, *28S* rRNA

## Abstract

The genus *Sarcocystis* is an abundant group of Apicomplexa parasites found in mammals, birds, and reptiles. These parasites are characterised by the formation of sarcocysts in the muscles of intermediate hosts and the development of sporocysts in the intestines of definitive hosts. The identification of *Sarcocystis* spp. is usually carried out in carcasses of animals, while there is a lack of studies on the detection of *Sarcocystis* species in blood samples. In the current study, blood samples of 214 yellow-necked mice (*Apodemus flavicollis*) and 143 bank voles (*Clethrionomys glareolus*) from Lithuania were examined for *Sarcocystis*. The molecular identification of *Sarcocystis* was carried out using nested PCR of *cox1* and *28S* rRNA and subsequent sequencing. *Sarcocystis* spp. were statistically (*p* < 0.01) more frequently detected in the bank vole (6.3%) than in yellow-necked mice (0.9%). The analysed parasites were observed in four different habitats, such as mature deciduous forest, bog, natural meadow, and arable land. Three species, *Sarcocystis funereus*, *Sarcocystis myodes*, and *Sarcocystis* cf. *glareoli* were confirmed in the bank vole, whereas only *Sarcocystis myodes* were found in yellow-necked mice. The obtained results are important in the development of molecular identification of *Sarcocystis* parasites in live animals.

## 1. Introduction

*Sarcocystis* (Apicomplexa: Sarcocystidae) is a genus of intracellular parasites that were first discovered in 1843 by F. Miescher in the muscles of the house mouse (*Mus musculus*) [1]. All *Sarcocystis* species use two hosts to complete their life cycle. Hosts of *Sarcocystis* species are usually determined by a prey–predator ecological relationship [2]. Intermediate hosts are infected by consuming food or water contaminated with mature sporocysts of *Sarcocystis* spp. After intake, sporozoites are released from the intestine of the host and enter the bloodstream where the schizogony takes place. Schizogony consists of several stages while the number of generations and the type of host cell varies based on *Sarcocystis* species [1,3]. Asexual reproduction results in the formation of sarcocysts in muscle tissues or CNS [1,4,5]. Definitive hosts become infected through the ingestion of tissues containing mature sarcocysts. Upon ingestion, sexual reproduction occurs within the small intestines of the definitive host. After the sporulation of oocysts, sporocysts are released into the environment together with the faecal matter [1,6].

Some *Sarcocystis* species are pathogenic to intermediate hosts, whereas in most cases they are not hazardous to definitive hosts [1]. These parasites are often found in livestock and annually cause losses in the animal husbandry industry [7]. Rodents are important for the transmission of various diseases [8]. However, limited data exists regarding the pathogenic *Sarcocystis* species that utilize rodents as intermediate hosts. *Sarcocystis glareoli* and *Sarcocystis microti*, which were previously assigned to the genus *Frenkelia*, form cysts in the brain of various rodent species [9]. The latter two *Sarcocystis* species are transmitted through the common buzzard (*Buteo buteo*) and potentially other members of the genus *Buteo* [10]. Additionally, *Sarcocystis singaporensis* has been used as a biological control agent against rats in a variety of agroforestry and agricultural environments [11].

To date, over 40 different *Sarcocystis* species have been identified in rodents. However, due to the low level of research on different rodent species, it is suggested that the true number of *Sarcocystis* spp. in these hosts is higher [12,13]. In recent years, several new *Sarcocystis* species have been described in rodents [14,15,16]. It should be noted that *Sarcocystis* spp. are most thoroughly examined in the house mouse (*Mus musculus*) and brown rat (*Rattus norvegicus*). However, data on the prevalence and richness of *Sarcocystis* species infecting wild mice and voles is limited.

Only some species of *Sarcocystis* produce sarcocysts that are visible to the naked eye, while the cysts of the other species of this genus are microscopic. The use of light or an electron microscope allows the differentiation of *Sarcocystis* species based on the size, and shape of the sarcocysts, their wall structure, and the morphometric features of bradyzoites that are located inside the cysts [16,17,18]. Nonetheless, the microscopical characterization and the isolation of sarcocysts from host tissues require specific competencies [19]. Additionally, the detection of morphologically similar *Sarcocystis* species in tissues of closely related intermediate hosts complicates microscopical analysis [1,20]. Therefore, the list of known *Sarcocystis* species is revised and new species are described by using combined morphological and molecular methods [21]. Currently, ribosomal RNA genes (*28S* rRNA or *18S* rRNA), internal spacer region 1 (*ITS1*), and mitochondrial cytochrome oxidase 1 (*cox1*) are mostly used for *Sarcocystis* species identification [12]. The selection of the genetic regions for the identification of the parasites depends on its host taxonomic group as it has been shown that *Sarcocystis* species co-evolved with their hosts [22].

To date, *Sarcocystis* parasites were mainly examined in animal carcasses by the aforementioned methods [23]. *Sarcocystis* species identification in blood would allow the detection of the parasite in living organisms. Conventional immunological methods are poorly suitable for the separation of *Sarcocystis* species due to the difficulties in the generation of species–specific antibodies [1,24]. Therefore, DNA analysis methods are appropriate for the diagnosis of *Sarcocystis* spp. in blood samples [25]. The first attempt to use molecular methods in blood samples of llamas instead of muscle tissues for the identification of *Sarcocystis* species was conducted in 2016 in Argentina [26]. A year later, the first known attempt at using the blood of rodents to identify *Sarcocystis* spp. was carried out in Japan. Even though the number of screened samples was small, *Sarcocystis* species were successfully detected in some of them [27]. In the following years, two unrelated studies were carried out in Nigeria and Turkey in which researchers successfully identified the DNA of *Sarcocystis* species in the blood samples of rodents [28,29].

An increasing amount of data indicates that *18S* rRNA is unsuitable for distinguishing sequences of related *Sarcocystis* species [17,21,30,31]. A recent study indicates that amplification of *ITS1* sequences from *Sarcocystis* spp. in rodents proves to be challenging [22]. Consequently, *28S* rRNA and *cox1* genetic regions were used for our study. Low concentrations of *Sarcocystis* species DNA in the bloodstream limit the use of conventional PCR methods. Thus, the nested PCR approach was applied in the current study to generate enough of the amplified products.

In 2016–2017, research was carried out to determine if rodents in Lithuania are infected with blood parasites such as *Babesia* spp., *Trypanosoma* spp., and *Hepatozoon* spp. [32]. Blood samples collected by Baltrūnaitė et al. [32] and additional samples gathered between 2018 and 2019 were employed in this investigation. For this study, we selected two of the most common and abundant rodent species in Lithuania, the yellow-necked mouse (*Apodemus flavicollis*) and the bank vole (*Clethrionomys glareolus*). These rodents dominate in forests but are also frequent in other habitats [33]. Thus, the objective of our work was to determine the prevalence of *Sarcocystis* spp. and to molecularly identify parasite species in the blood samples of *A. flavicollis* and *C. glareolus* from Lithuania.

## 2. Materials and Methods

### 2.1. Blood Sample Collection

**Figure 1:**
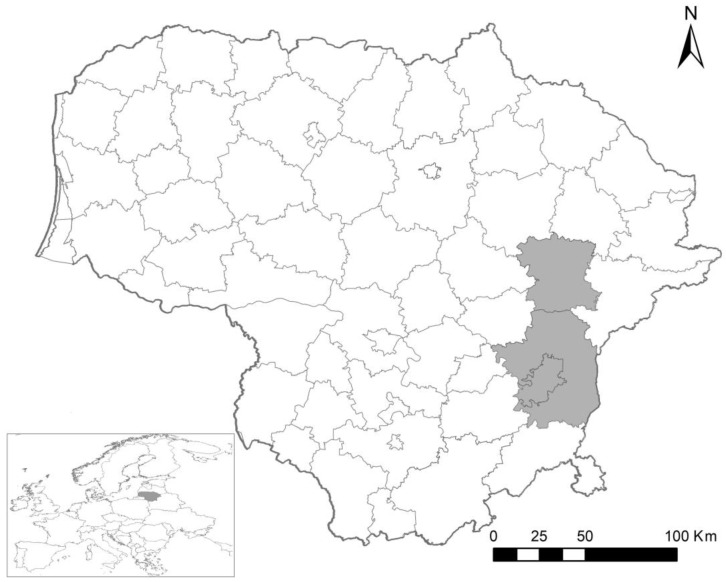
Small mammal trapping areas in Lithuania during the 2016–2019 period. Sampled districts are marked in grey.

The study was carried out in Lithuania, Vilnius, and Molėtai districts, in 2016–2019 (May–November) (Figure 1).

A highly fragmented landscape composed of various open and forest habitats was typical of the study area. The habitats ranged from the ones not disturbed by human activity (e.g., natural meadows, bogs) to the intensively used (e.g., arable land). During the four-year period, a total of 357 blood samples were collected, with 214 of the samples belonging to *A. flavicollis* and 143 to *C. glareolus*. Small mammals were trapped in various habitats, namely mature deciduous forests, and mixed forests, planted young forests, bogs, natural and shrubby meadows, and arable land. Sherman live traps were baited with pieces of bread soaked in sunflower oil and bedding material. Traps were set in the evening and checked early in the morning. Live animals were humanely killed by cervical dislocation. Blood from the heart was collected in SET buffer immediately after death and stored in the freezer at −20 °C until further molecular analysis [34]. Trapped rodents were identified to species and weighed. The sex and age (juveniles, sub-adults, adults) were determined during dissection. The age was based on the atrophy of the thymus gland and the status of reproductive organs [35,36,37].

### 2.2. Molecular Analysis of A. flavicollis and C. glareolus Blood Samples

Total DNA extraction was performed using standard ammonium acetate protocol [38]. Nested PCR of *28S* rRNA and *cox1* was employed to detect the presence of *Sarcocystis* spp. in the blood samples of *A. flavicollis* and *C. glareolus*. External primer pair of SF1/SR5 and internal primer pair of SgraucoF1/SgraucoR1 were used for the amplification of *cox1* sequences, while the Sgrau281/Sgrau282 and Sgrau283/Sgrau284 primer pairs were employed for the *28S* rRNA [22].

The first PCR reaction was carried out in 25 µL reaction volume, containing 12.5 µL of Dream Taq PCR Master Mix (Thermo Fisher Scientific Baltics, Vilnius, Lithuania), 7.5 µL of nuclease-free water, 0.5 µM of each primer and 4 µL of the template DNA. For the second step of the PCR, 2 µL of the products obtained from the initial PCR were utilized. The rest of the reaction volume contained 12.5 µL of Dream Taq PCR Master Mix (Thermo Fisher Scientific Baltics, Vilnius, Lithuania), 9.5 µL nuclease-free water, and 0.5 µM of each primer. Water was used as the negative control instead of the DNA template for both steps of the nested PCR. DNA extracted from *Sarcocystis myodes* cysts [16] served as positive control in this study.

The amplification of the first PCR was conducted using the following program: initial denaturation at 95 °C for 5 min, followed by 35 cycles of 45 s at 95 °C, 55 s at 59–61 °C (depending on the annealing temperature of primers), followed by 65 s at 72 °C and the final extension for 7 min at 72 °C. The second round of the PCR was performed as follows: initial denaturation at 95 °C for 5 min, followed by 35 cycles of 35 s at 95 °C, 45 s at 59 °C, 55 s at 72 °C and ended with the final extension at 72 °C for 7 min. After completion of each PCR step products were visualized on 1% agarose gels using electrophoresis.

All the positive samples were purified with alkaline phosphatase FastAP and exonuclease ExoI (Thermo Fisher Scientific Baltics, Vilnius, Lithuania) according to the instructions of the manufacturer. The purified samples were sequenced with forward and reverse primers used for a second PCR step. Big-Dye^®^ Terminator v3.1 Cycle Sequencing Kit (Thermo Fisher Scientific, Vilnius, Lithuania) and the 3500 Genetic Analyzer (Applied Biosystems, Foster City, CA, USA) were employed for Sanger sequencing. All the acquired chromatograms were pure, without double peaks. Nucleotide BLAST (http://blast.ncbi.nlm.nih.gov/, accessed on 27 January 2024) tool was utilized to compare sequences detected in this study with *Sarcocystis* spp. sequences in the NCBI GenBank database.

Multiple alignments of *cox1* and *28S* rRNA sequences were created with the help of the MUSCLE algorithm implemented in MEGA7 [39]. The alignment of *cox1* contained 22 taxa and 619 nucleotide positions without gaps, whilst *28S* rRNA alignment consisted of 27 taxa and 803 nucleotide positions including gaps. The selection of evolutionary models and generation of phylogenetic trees was performed while using TOPALi v2.5 [40]. The Bayesian method was applied to uncover phylogenetic relationships. The F81 + G and HKY + G nucleotide substitution models were chosen for *cox1* and *28S* rRNA analysis. *Toxoplasma gondii* was chosen as the outgroup in the generation of phylogenetic trees. The analyses were carried out in two runs, using one million generations with a sample frequency of 10 and 25% burn-in value. The *28S* rRNA and *cox1* sequences of the *Sarcocystis* spp. from the present study are available in GenBank under the accession numbers PP350819–PP350829 and PP358794–PP358804.

### 2.3. Statistical Analysis

Quantitative parasitology 3.0 software was utilized for statistical tests [41]. Sterne’s exact method was applied to determine a 95% confidence interval (CI) for the detection rates of *Sarcocystis* spp. Fisher’s exact test was utilized to assess the statistical significance of the differences comparing the prevalence of *Sarcocystis* spp. observed in the samples of *A. flavicollis* and *C. glareolus*.

## 3. Results

### 3.1. Detection Rates of Sarcocystis spp. in the Blood Samples of A. flavicollis and C. glareolus

For this study, 143 samples of *C. glareolus* were collected from traps in seven different habitats, such as mature deciduous forests and mixed forests, planted young forests, bogs, natural and shrubby meadows, and arable land (Table 1). The number of specimens caught in each habitat ranged from 8 to 34. Molecular examination of blood samples revealed the presence of *Sarcocystis* spp. in individuals collected from four different habitats: arable land, bog, mature deciduous forest, and natural meadow. In the latter habitats, the rate of positive samples ranged from 11.11% (95% CI = 2.02–33.02) to 12.50% (95% CI = 0.64–50.00). Differences in detection rates of *Sarcocystis* spp. were statistically insignificant when comparing the habitats tested (*p* > 0.05). The overall frequency of *Sarcocystis* spp. in the investigated blood samples of *C. glareolus* was 6.29% (95% CI = 3.19–11.45).

Additionally, 214 individuals of *A. flavicollis* were caught in seven different habitats, including mature deciduous forests and mixed forests, planted young forests, bogs, natural and shrubby meadows, and arable land (Table 2). In all but two habitats, between 29 and 63 individuals were trapped. In the remaining two habitats, planted young forest and bog, only five and eight individuals were collected, respectively. Molecular analysis of blood samples revealed the presence of *Sarcocystis* spp. in individuals collected only in arable land 3.17% (95% CI = 0.57–10.87). Statistically significant differences in detection rates of *Sarcocystis* spp. across the analysed habitats were not observed (*p* > 0.05). The detection frequency was significantly lower in *A. flavicollis* (0.93%; 95% CI = 0.17–3.40) compared to *C. glareolus* (*p* = 0.009).

### 3.2. Molecular Characterisation of Sarcocystis spp. in A. flavicollis and C. glareolus

In this study, 11 isolates were successfully characterized within partial *cox1* and *28S* rRNA. The length of the analysed *cox1* sequences was 619 bp, while *28S* rRNA sequences ranged between 721 bp and 735 bp. At the *cox1* loci, sequences obtained in this study shared a similarity of 94.67–100% amongst themselves, while at the *28S* rRNA loci the similarity was 87.25–100%. Thus, *28S* rRNA was more variable compared with the *cox1* for the *Sarcocystis* spp. detected. Based on the molecular analysis, three *Sarcocystis* species (*Sarcocystis funereus*, *S. myodes* and *Sarcocystis* cf. *glareoli*) were identified (Table 3).

According to the analysis of *cox1* gene fragments, the sequences of *S. myodes* demonstrated 99.84–100% similarity compared to the sequences of this species deposited in the NCBI GenBank. Additionally, fragments acquired from the amplification of the *28S* rRNA loci demonstrated intraspecific similarity of 99.46–100% in comparison with other *S. myodes* sequences. Based on *28S* rRNA, the sequences of *S. myodes* showed the highest similarity to those of *Sarcocystis ratti* and *Sarcocystis* sp. Rod1 (96.46–97.82%).

The second detected *Sarcocystis* species (isolates LTKrCgla7A and LTKrCgla25C) exhibited the highest genetic similarity with two *Sarcocystis* species (*S. glareoli* and *S. microti*) previously classified in the genus *Frenkelia* and *Sarcocystis jamaicensis* (Table 3). However, *cox1* sequences of *S. glareoli* and *S. microti* are yet to be established. The analysed *Sarcocystis* isolate displayed 100% similarity with *S. jamaicensis* within the *cox1*. Based on *28S* rRNA, this organism differed from *S. jamaicensis* by two single nucleotide polymorphisms (SNPs) and by a single indel (insertion/deletion) from *S. glareoli*. Therefore, as nucleotide substitutions have more value for species divergence than indels [42], this *Sarcocystis* organism was denoted as *S*. cf. *glareoli*.

The last found species in this investigation was recently described as *S. funereus* [43]. At *28S* rRNA, obtained sequences shared 99.72–100% similarity with those of *S. funereus*, 95.60% with those of *Sarcocystis lari*, and 95.25% with those of *Sarcocystis strixi. Cox1* sequences of *S. funereus* have not been identified to date. Thus, based on this gene, sequences of *S. funereus* obtained in our work demonstrated the highest similarity with those of *S. lari*, *Sarcocystis lutrae*, and *S. strixi* (99.35–99.52%).

### 3.3. Phylogenetic Analysis of Identified Sarcocystis Species

**Figure 2:**
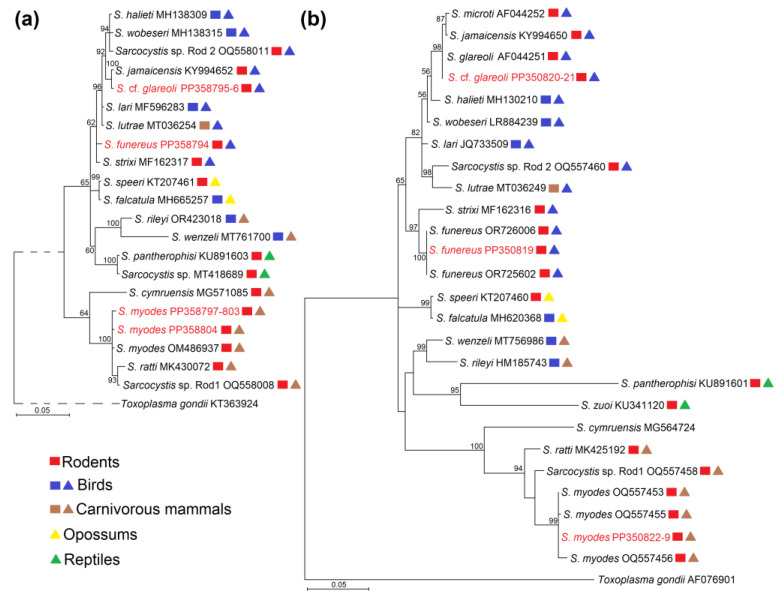
The phylogenetic relationships of the *Sarcocystis* spp. identified in blood samples of *C. glareolus* and *A. flavicollis* based on *cox1* (**a**) and *28S* rRNA (**b**) sequences. Phylograms were generated using Bayesian methods, scaled according to the branch length, and rooted on *Toxoplasma gondii*. The posterior probability support values are indicated next to branches, and GenBank accession numbers are given behind the species name. The sequences of three *Sarcocystis* species (*S. funereus*, *S. myodes*, and *S*. cf. *glareoli*) obtained in this work are shown in red. The coloured rectangles and triangles indicate the identified or presumed intermediate hosts and definitive hosts of *Sarcocystis* species, respectively.

The phylogenetic analyses based on two analysed loci (*cox1* and *28S* rRNA) resulted in longer branches of phylograms when using *28S* rRNA sequences (Figure 2). In the *28S* rRNA phylogenetic tree, sequences of three *Sarcocystis* species detected in this work were grouped with other isolates of the corresponding species, thus confirming the identification of the species studied. In both phylograms, two of the examined species, *S. funereus* and *S*. cf. *glareoli* were placed together with *Sarcocystis* species employing rodents, birds, and carnivorous mammals as their intermediate hosts and birds of their identified or presumed definitive hosts according to phylogenetic studies. Meanwhile, *S. myodes* was grouped into several *Sarcocystis* species using rodents and carnivorous mammals as their defined or proposed definitive hosts. Based on sequences of more variable *28S* rRNA, *S*. cf. *glareoli* was most closely related to *S. jamaicensis* and *S. microti*, whereas *S. funereus* was sister species to *S. strixi* and finally *S. myodes* was grouped with *S. ratti* and *Sarcocystis* sp. Rod1.

## 4. Discussion

### 4.1. Prevalence of Sarcocystis spp. in Rodents

This study is the first documented attempt at investigating blood samples of *C. glareolus* and *A. flavicollis* for the detection of *Sarcocystis* species. In prior Lithuanian studies, sarcocysts were found in the muscles of several rodent species, including the brown rat (*Rattus norvegicus*), black rat (*Rattus rattus*), the bank vole, common vole (*Microtus arvalis*), tundra vole (*Alexandromys oeconomus*), field vole (*Microtus agrestis*), yellow-necked mouse, and striped field mouse (*Apodemus agrarius*) [16,44,45,46,47].

Specifically, the reported prevalence of *Sarcocystis* parasites in *C. glareolus* in Lithuania varies from 1.34% to 14.38% [16,22,45,48,49]. In this study, DNA of *Sarcocystis* spp. was detected in 6.29% (95% CI = 3.19–11.45) of *C. glareolus* blood samples, which is consistent with results from prior studies using muscle samples in Lithuania. Similar detection rates were observed in the Czech Republic and Finland, with *Sarcocystis* spp. detection in *C. glareolus* being relatively low, at 1.46% and 6%, respectively [50,51]. Nevertheless, studies on other vole species highlight significantly higher *Sarcocystis* species prevalence. In China, a study found that 25% of large oriental voles (*Eothenomys miletus*) were infected with *Sarcocystis* species [14]. Similarly, in Argentina, wild Cricetidae species exhibited a prevalence of 16.08% [12], while in Japan, 16.85% of Bedford’s red-backed voles (*Clethrionomys rufocanus bedfordiae*) were infected [52]. Moreover, research conducted in the Netherlands on *M. arvalis* reported seasonal variations in *Sarcocystis* spp. prevalence among voles, ranging from 6% to 33% [53].

Formerly, species producing cysts in the muscle tissues were classified under the genus *Sarcocystis* [54], while parasite species forming cysts in the brains of small mammals were placed under the genus *Frenkelia* [55] due to differences in sarcocyst morphology and its location. However, the merging of these genera has been proposed by several authors based on phylogenetic studies [9,56]. In Lithuania, it was reported that the prevalence of *Frenkelia* spp. in *C. glareolus* was 21.11% [57]. By comparison, in the Czech Republic, the prevalence of *Frenkelia* spp. was 38.5% [51], while in Germany it ranged from 10.34% to 55.93% [58,59], and in France, it varied from 1.02% to 48.14% [60,61].

Investigations on the prevalence of *Sarcocystis* spp. and *Frenkelia* spp. in *A. flavicollis* are notably scarce. In Lithuania, previously reported detection rates of *Sarcocystis* parasite in muscles ranged from 0% to 0.84%, consistent with the findings of this investigation, which reported a prevalence of 0.93% (95% CI = 0.17–3.40) [22,45,48,49]. In studies conducted in the Czech Republic and Spain, no *Sarcocystis* species were identified in the muscle samples of mice [51,62]. Taking into consideration other species of the genus *Apodemus*, higher infection rates of *Sarcocystis* spp. were recorded, namely in the large Japanese field mouse (*Apodemus speciosus*) and the small Japanese field mouse (*Apodemus argenteus*), the prevalence of *Sarcocystis* spp. was 18.99% and 21.43%, respectively [52]. In a previous Lithuanian study, *Frenkelia* spp. was absent in all *A. flavicollis* specimens [57]. Meanwhile, the prevalence of *Frenkelia* spp. in *A. flavicollis* from the Czech Republic was 2.40% [51], and in Germany, it ranged from 0.45% [59] to 8.54% (95% CI = 4.2–16.6) [58]. In summary, studies carried out in Lithuania, Germany, and the Czech Republic indicate that *Sarcocystis* spp. is more commonly found in *C. glareolus* than in *A. flavicollis* (present study, [51,57,58,59]).

### 4.2. Detection of Sarcocystis spp. in Blood Samples of Intermediate Hosts

During this investigation, blood samples were used to determine the prevalence and *Sarcocystis* spp. richness in Lithuania for the first time. Additionally, prior documented research on blood samples to identify *Sarcocystis* spp. has been conducted in Argentina, Japan, Turkey, Nigeria, and Australia, indicating precedence for such studies [25,26,27,28,29,63]. Three separate studies utilized rodent blood samples for investigation, successfully detecting *Sarcocystis* spp. in the blood of grey-sided vole (*Myodes rufocanus*), wood mouse (*Apodemus sylvaticus*), black rat and brown rat [27,28,29]. Remarkably, the prevalence of *Sarcocystis* spp. in *M. rufocanus* from Japan was relatively high, reaching 16.67%; nonetheless, this observation could be attributed to the small sample size (*n* = 6) that was examined [27]. Blood samples collected in Nigeria and Turkey from *A. sylvaticus*, *R. rattus*, and *R. norvegicus* exhibited an overall prevalence of *Sarcocystis* parasites ranging between 0.19% and 2.1% [28,29], aligning with the low prevalence rates reported in our study.

Regrettably, in this study, it was not possible to compare data from samples collected from blood and muscle tissues of the same individuals. Despite this, a study in Lithuania was carried out recently to identify *Sarcocystis* spp. in the muscle samples of rodents from commercial orchards [22]. Due to the sheer volume of the samples, the authors pooled muscle samples of the rodents that belonged to the same species, digested them, and identified parasite species using nested PCR and sequencing. In the study, the prevalence of *Sarcocystis* spp. in *A. flavicollis* was recorded as 0.84% (95% CI = 0.15–2.75), a finding that correlates closely with the results of our investigation, which showed a detection rate of 0.93% (95% CI = 0.17–3.40). Meanwhile, the rate of *Sarcocystis* infection in the muscles of *C. glareolus* was 1.34% (95% CI = 0.08–6.43) [22], showing a lower detection rate compared to our study, in which the rate was 6.29% (95% CI = 3.19–11.45). The variance in the results may be due to the different stages of *Sarcocystis* spp. infection, distinct sample collection locations, and potential seasonal effects [3,53]. Findings from a study conducted on camelids suggest that the DNA of *Sarcocystis* spp. can be detectable in the blood during the initial phases of the infection but become undetectable once encystment occurs [25].

The identification of three *Sarcocystis* species in this study highlights the significance of utilizing blood samples from intermediate hosts as a valuable method for examining both *Sarcocystis* prevalence and species diversity. This is the first report of *S. funereus* in *C. glareolus* from Lithuania, although molecular studies of muscle tissues were conducted before [16,22]. Although the molecular method used in this investigation is not suitable for the detection of *Sarcocystis* species coinfections. To identify more than one *Sarcocystis* species in a single sample, other methods, e.g., cloning, might be used [64].

### 4.3. Characteristics of Sarcocystis spp. in A. flavicollis and C. glareolus

Our molecular analysis showed the presence of three *Sarcocystis* species in *A. flavicollis* and *C. glareolus* from Lithuania. Among these species, *S. myodes* was only recently identified and characterised in the muscles of a single *C. glareolus* specimen [16]. Subsequently, sarcocysts of *S. myodes* have been detected in other intermediate hosts, such as *A. flavicollis*, *A. agrarius*, and *M. arvalis* [22]. Morphological analysis revealed sarcocysts of *S. myodes* to be microscopic (600–3000 × 70–220 µm) with a thin (~1 µm), smooth cyst wall lacking visible protrusions [16]. The characteristics of *S. myodes* sarcocysts closely resemble those of *S. microti* and *S. glareoli*. However, the latter species are exclusively found in the brains of small mammals [10,65]. Molecular analysis data on *18S* rRNA and *28S* rRNA loci demonstrates reliable differences between the three species [16,47,66]. While *S. glareoli* and *S. microti* utilise buzzards (*Buteo* spp.) as their definitive host [16,65], the primary host of *S. myodes* remains speculative due to insufficient data. Nevertheless, phylogenetic analysis suggests a potential association of *S. myodes* with predatory mammals [22].

Additionally, *S. myodes* shares structural similarities in its sarcocyst wall with *S. cernae* found in the common vole, as well as with *Sarcocystis montanaensis* discovered in the prairie vole (*Microtus achrogaster*), the long-tailed vole (*Microtus longicaudus*), and the eastern meadow vole (*Microtus pennsylvanicus*) [67,68,69]. However, the bradyzoites of *Sarcocystis cernae* (8–9 × 2–2.5 µm) [67] and *S. montanaensis* (9.8–12.2 × 2.2–4.3 µm) [68] differ from those of *S. myodes* (9.6–12.0 × 3.1–4.6 μm) [16]. A variety of snake species serve as definitive hosts in the life cycle of *S. montanaensis*, while *S. cernae* exclusively relies on the common kestrel (*Falco tinnunculus*) according to transmission experiments [68,69]. Thus, *S. myodes* differs from other *Sarcocystis* species found in different vole species, as determined through morphological, genetic, and phylogenetic analyses [16]. In addition, *S. myodes* displays sequence resemblance to *S. ratti*, found in the muscles of black rats across four loci (*18S* rRNA, *28S* rRNA, *cox1*, *ITS1*) [16,47]. Morphologically, both parasite species share similar sarcocyst size, shape, and wall structure, although *S. myodes* exhibits longer bradyzoites compared to those of *S. ratti* (7.5–9.3 × 3.9–4.8 µm) [47].

During our study, one of the *Sarcocystis* organisms was identified tentatively as *S*. cf. *glareoli*. The lack of molecular data for the *S. glareoli* species, which was once placed under the genus *Frenkelia*, causes a significant challenge in the identification of this species. Currently, only sequences of the *18S* rRNA and *28S* rRNA genes have been obtained [9,10]. Apart from *cox1*, which was used in numerous studies [12,16,21,47,70,71], additional genetic markers, such as *ITS1*, mitochondrial cytochrome b (*cytb*), complete *ITS1*–*5.8S* rRNA–*ITS2* region, two apicoplast genes—RNA polymerase beta subunit (*rpoB*) and caseinolytic protease C (*clpC*) demonstrate promising prospects for improved discrimination of *Sarcocystis* spp. in small mammals [47,66,72,73].

The last *Sarcocystis* species identified in our investigation is *S. funereus*, recently detected in the Tengmalm’s owl (*Aegolius funereus*) population in Finland [43]. Formerly identified as *Sarcocystis* sp. isolate Af1, this species lacked comprehensive data concerning its intermediate host and sarcocyst structure, despite characterisation using four genetic loci (*ITS1*, *cox1*, *18S* rRNA, and *28S* rRNA) [74]. However, *S. funereus* has been differentiated from other *Sarcocystis* species primarily through the utilization of the *28S* rRNA and *ITS1* region [43]. The latter region demonstrates superior sensitivity in discerning genetic variances among *Sarcocystis* species with birds and carnivores as intermediate hosts [74,75,76]. While the natural intermediate host remains elusive, experimental findings proposed *C. glareolus* as a potential candidate [77]. Notably, *A. funereus* exhibits a dietary preference for voles, with bank voles comprising more than 40% of its diet [78]. Our investigation provides additional evidence supporting the hypothesis that *C. glareolus* serves as the natural intermediate host for *S. funereus*.

## 5. Conclusions

Our comprehensive study marks a significant advance in the research of *Sarcocystis* spp. by using blood samples for the investigation of these parasites in two rodent species, *A. flavicollis* and *C. glareolus*. The findings revealed varying frequencies of *Sarcocystis* spp. across habitats, with no statistically significant differences observed in detection rates. Moreover, prior investigations carried out in Lithuania, Germany, and the Czech Republic indicate that *C. glareolus* tends to have higher rates of *Sarcocystis* spp. detection compared to *A. flavicollis*, which is consistent with the prevalence rates observed in the current study. Additionally, molecular analysis revealed three distinct *Sarcocystis* species—*S. myodes*, *S. cf. glareoli*, and *S. funereus*, with the latter being identified in Lithuania for the first time. Thus, blood samples can be successfully used for the studies of *Sarcocystis* spp. richness in small mammals.

## Figures and Tables

**Table 1:** Detection rates of *Sarcocystis* spp. in blood samples of *C. glareolus*.

Habitat	Trapped	Infected (%)	*Sarcocystis* Species
Mature deciduous forest	18	2 (11.11%)	*S. myodes*
Mature mixed forest	33	0	-
Planted young forest	10	0	-
Bog	8	1 (12.50%)	*S. myodes*
Natural meadow	34	4 (11.76%)	*S. myodes* and *S. funereus* *
Shrubby meadow	22	0	-
Arable land	18	2 (11.11%)	*S*. cf. *glareoli*
Total	143	9 (6.29%)	

* *Sarcocystis myodes* was detected in three samples and *S. funereus* was detected in a single sample.

**Table 2:** Detection rates of *Sarcocystis* spp. in blood samples of *A. flavicollis*.

Habitat	Trapped	Infected
Mature deciduous forest	33	0
Mature mixed forest	45	0
Planted young forest	5	0
Bog	8	0
Natural meadow	31	0
Shrubby meadow	29	0
Arable land	63	2 (3.17%) *
Total	214	2 (0.93%)

* Only one species *S. myodes* was confirmed by molecular methods.

**Table 3:** *Sarcocystis* species identified in this study, and the percentage of similarity compared with the most closely related species.

Genetic Similarity with the Most Closely Related Species by Different Genes			
*Sarcocystis* species	*cox1*	*Sarcocystis* species	*28S rRNA*
*S. myodes* (619 bp)	*S. myodes* (99.84–100%), *Sarcocystis* sp. Rod1 (99.52–99.68%), *S. ratti* (99.03–99.19%)	*S. myodes* (735 bp)	*S. myodes* (99.46–100%), *Sarcocystis* sp. Rod1 (97.82%), *S. ratti* (96.46%)
*S*. cf. *glareoli* (619 bp)	*S. jamaicensis* (100%), *Sarcocystis* sp. SCMW1 (99.68%), *S. lutrae*, *S. corvusi*, *S. columbae*, *S. halieti*, *S. lari* (99.52%), *S. wobeseri*, *S. cornixi*, *Sarcocystis* sp. ex *Accipiter cooperi*, *Sarcocystis* sp. Rod2 (99.35%), *S. turdusi* (99.19%), *S. caninum*, *S. arctica*, *S. strixi*, *S.* cf. *strixi* (99.03%)	*S*. cf. *glareoli* (726 bp)	*S. glareolid* (99.86%), *S. jamaicensis* (99.72%), *S. microti* (98.62%)
*S. funereus* (619 bp)	*S. strixi* (99.52%), *S. lutrae*, *S. lari* (99.35%), *Sarcocystis* sp. Ex *Accipiter cooperi*, *Sarcocystis* sp. SCMW1 (99.19%), *S. corvusi*, *S. columbae*, *S. halieti* (99.03%)	*S. funereus* (721 bp)	*S. funereus* (99.72–100%), *S. lari* (95.60%), *S. strixi* (95.25%)

The lengths of the analysed fragment are indicated in parentheses under the name of *Sarcocystis* species.

## Data Availability

The *28S* rRNA and *cox1* sequences of *S. funereus*, *S. myodes* and *S*. cf. *glareoli* are available via the NCBI GenBank database under accession numbers PP350819–PP350829 and PP358794–PP358804.
